# Outcomes From Routine Use of Intraoperative Cholangiogram in Laparoscopic Cholecystectomy: Factors Predicting Benefit From Selective Cholangiography

**DOI:** 10.7759/cureus.12555

**Published:** 2021-01-07

**Authors:** Akinfemi Akingboye, Fahad Mahmood, Marriam Ahmed, Kishan Rajdev, Osama Zaman, Harvinder Mann, Sujeewa C Sellahewa

**Affiliations:** 1 General and Colorectal Surgery, Russells Hall Hospital, Dudley, GBR

**Keywords:** intra-operative cholangiogram, choledocholithiasis, cbd stone, ercp, laparoscopic cholecystectomy, deranged liver function tests

## Abstract

Background and objective

Laparoscopic cholecystectomy is used for the treatment of symptomatic gallstones. Intraoperative cholangiogram (IOC) is used to diagnose common bile duct (CBD) stones. There is controversy surrounding routine vs selective use of IOC based on clinical, biochemical and ultrasound criteria. The aim of this study was to evaluate the outcomes from routine IOC and its utility in laparoscopic cholecystectomy.

Materials and methods

This was a UK-based single-centre retrospective study evaluating the outcomes from IOC for all laparoscopic cholecystectomies performed between May 2014 and February 2020. All adult patients undergoing elective, semi-elective or emergency operations were included. Demographics, biochemistry as well as radiological and endoscopic investigations were analysed. IOC was performed using a standardised technique and was interpreted by a single surgeon.

Results

A total of 744 out of 804 patients underwent IOC. The median age of the cohort was 51 years (SD: ±17.5); there were 468 females (62.9%) and 276 males (37.1%). Filling defects were identified in 43/744 (5.8%) patients, with 23/43 having stone extraction via endoscopic retrograde cholangiopancreatography (ERCP). Logistic regression analysis identified alkaline phosphatase (ALP) as a predictor of filling defects in IOC (OR: 1.003; 95% CI: 1.001-1.005, p=0.015).

Conclusion

Based on our findings, the routine use of IOC during laparoscopic cholecystectomy is safe and effective. Preoperative clinical, radiological and biochemical parameters apart from ALP have a limited role in predicting the diagnostic yield of IOC.

## Introduction

The incidence of gallstones is estimated to be 6-15% with a further 10-15% of these patients presenting with common bile duct (CBD) stones [1]. These in turn can lead to complications including cholangitis, pancreatitis and obstructive jaundice. Furthermore, the initial diagnosis of CBD stones is performed through a combination of assessment of liver function tests (LFTs) [gamma-glutamyltransferase, alkaline phosphatase (ALP), alanine aminotransferase (ALT), aspartate aminotransferase (AST) and bilirubin] and trans-abdominal ultrasound (US) scan [1-3]. The negative predictive value for CBD stones for combined LFTs is 97% and 95% for a ductal diameter of 3-6 mm on US scan [2,3]. However, these techniques have low sensitivity and specificity, meaning further investigations including magnetic resonance cholangiopancreatography (MRCP), endoscopic ultrasound (EUS), endoscopic retrograde cholangiopancreatography (ERCP) or intraoperative cholangiogram (IOC) are often required.

IOC involves injecting a dye through the cystic duct during cholecystectomy, and it has a reported sensitivity and specificity of 99% for CBD stones [4,5]. There is an ongoing debate about the benefits of routine vs selective IOC in diagnosing CBD stones. Selective IOC is based upon specified clinical, biochemical and/or US findings as well as intraoperative anatomical distortion [6]. However, this may lead to CBD stones being missed with delayed presentation of patients in the acute setting. Moreover, routine IOC may be associated with false-positives as well as increased complication risk and operative time [7]. The aim of this study was to assess the utility of routine IOC in detecting CBD stones in a dedicated single upper gastrointestinal (GI) surgery unit.

## Materials and methods

The study was conducted as a retrospective review of prospectively maintained data at a single UK district hospital between May 2014 and February 2020. All cases were performed by a single surgeon. All adults (age of >18 years) undergoing laparoscopic cholecystectomy during the time period were deemed eligible. This included patients undergoing elective (planned procedure or >6 weeks after the presentation to emergency services), semi-elective (within six weeks of presenting as an emergency admission) and emergency (surgery on index emergency admission) operations.

The data collected included patient demographics, preoperative LFTs and preoperative and histological diagnoses. Furthermore, pre and postoperative investigations including US scan and MRCP results along with interventions including ERCP were ascertained. In addition, the data related to the length of stay, readmission and complications were collected.

IOC was performed using a standard technique with a cholangiogram catheter inserted through a cut opening in the cystic duct; 20 ml of Omnipaque™ 300 (GE Healthcare, Chicago, IL) was injected through the cholangiogram catheter followed by X-ray imaging of the biliary tree. Filling defects were confirmed by the operating surgeon with subsequent assessment and removal by ERCP to determine the presence of a stone. ERCP was performed within six weeks either pre or postoperatively. Failure of IOC was defined as the technical inability to perform the procedure, or decision by the operating surgeon to not perform due to challenging anatomy. The reasons for this were determined, including whether CBD stones were detected by other methods postoperatively.

Statistical analysis

Data were presented as mean ±standard deviation (SD), or as medians and ranges. Continuous variables were compared using the independent t-test, while categorical variables were compared using the chi-squared test. Logistic regression analyses were performed to identify factors independently associated with predicting the presence of CBD stones. This was performed using the SPSS Statistics software version 25 (IBM, Armonk, NY).

This study was registered with our local audit department as a quality improvement project (Audit/IRB number: GENSUR/2020/07). No ethical approval was required for this study from our local department.

## Results

A total of 804 records between 2014-2020 were analysed, of which 744 patients underwent IOC. The median age of our cohort was 51 years (SD: ±17.5); there were 468 females (62.9%) and 276 males (37.1%). All patients underwent preoperative investigations including US scans, haematological, biochemical and LFT assessment with some patients undergoing further investigations in the form of an MRCP; 84/744 patients had a bilirubin level above 20 mmol/L; 86/744 had ALP above 144 IU/L; and 148/744 had ALT above 56 IU/L. The median delay to surgery was 131 days (SD: ±346). The median length of stay was one day (range: 1-28 days) with no mortality reported at 30- and 90-day follow-ups; 37 patients (5%) re-attended with pain or complications including wound infection, collection and urinary retention. This further included one patient with a benign biliary stricture, although there were no bile leaks, intra-abdominal bile collections (biloma) or CBD injuries in our cohort of patients. No patients required a return to theatre or intensive care admission. A summary of the baseline demographics and preoperative LFTs are outlined in Table 1.

**Table 1:** Summary of the baseline demographics and preoperative liver function tests of patients undergoing laparoscopic cholecystectomy with intraoperative cholangiogram SD: standard deviation

Variable	Values
Age in years, mean ±SD	51 ±17.5
Gender	468 females (62.9%); 276 males (37.1%)
Bilirubin (3-20 mmol/L), mean ±SD	12.5 ±16 mmol/L
Alanine aminotransferase (7–56 IU/L), mean ±SD	58 ±96 IU/L
Alkaline phosphatase (44–147 IU/L), mean ±SD	106 ±99 IU/L

The majority of patients underwent laparoscopic cholecystectomy and IOC for biliary colic (53%) with the rest undergoing it for acute cholecystitis, gallstone pancreatitis, gallbladder polyps and gallbladder dyskinesia (Table 2). Furthermore, 419 (56.3%) patients were operated on in the elective setting, with 274 (36.8%) undergoing semi-elective procedures and 51 (6.9%) undergoing emergency operations during the index admission (Table 3).

**Table 2:** Indications for laparoscopic cholecystectomy and IOC IOC: intraoperative cholangiogram

Diagnosis	Frequency	Percentage
Acute cholecystitis	221	29.7
Biliary colic	394	53
Gallstone pancreatitis	111	14.9
Other (e.g., gallbladder polyp)	18	2.4

**Table 3:** Proportion of patients undergoing elective, semi-elective and emergency laparoscopic cholecystectomy + IOC with the proportion of filling defects on IOC for each group IOC: intraoperative cholangiogram

Urgency of surgery	Frequency	Overall percentage	Frequency (%) of OTC filling defect
Elective	419	56.3	24 (5.7%)
Semi-elective	274	36.8	13 (4.7%)
Emergency	51	6.9	6 (11.8%)

Of the 744 patients who underwent IOC, filling defects were observed in 43 (5.8%) patients. The frequency of filling defects according to the urgency of surgery is outlined in Table 3; 29/43 (67%) patients underwent postoperative ERCP, of which 23 had CBD stones extracted, giving a false-positive rate of 46.5% for IOC. The remaining 14/43 patients had either a EUS or MRCP performed, which did not confirm the presence of CBD stones, and hence no further intervention was performed. No patients underwent on-table CBD exploration. In addition, of the 43 patients with positive IOCs, 15/43 had abnormal LFTs (high bilirubin, ALP or both), 5/43 had dilated bile ducts evident on US scan and only 1/10 MRCPs performed showed a distinct filling defect in the CBD. This highlighted the variation in results obtained by different imaging modalities. 

Logistic regression analysis was performed to identify the factors predicting the likelihood of observing filling defects within IOC. Raised ALP was found to be significantly associated (OR: 1.003; 95% CI: 1.001-1.005, p=0.015) with a higher likelihood of observing filling defects on IOC (Table 4). No other parameters were found to be significantly associated with detecting filling defects on IOC. 

**Table 4:** Logistic regression analysis to identify variables predicting filling defects on IOC during laparoscopic cholecystectomy Only raised alkaline phosphatase was significantly predictive of finding a filling defect *Statistically significant ALT: alanine aminotransferase; ALP: alkaline phosphatase

Variable	Odds ratio	95% CI	P-value
Age	1.014	0.995–1.034	0.142
Gender	0.899	0.468–1.727	0.749
Semi-elective surgery	1.475	0.708–3.072	0.299
Emergency surgery	1.378	0.372–5.110	0.631
Bilirubin	0.993	0.974–1.013	0.503
ALT	1.002	0.999–1.005	0.259
ALP	1.003	1.001–1.005	0.015*

Of note, 183 patients underwent preoperative MRCP, of whom 16 (8.7%) had CBD stones reported; 6/16 had preoperative ERCP, whereas CBD stones were only confirmed in one of the remaining 10 patients by IOC, who went on to have a postoperative ERCP. Of the 167 patients in whom preoperative MRCP did not identify a CBD stone, eight had filling defects identified on IOC with four of these patients requiring postoperative ERCP. The justification for performing IOC in those who had preoperative MRCP or ERCP was the delay to surgery. Some patients not having intervention despite filling defects on IOC was due to the ambiguous nature of the filling defects on IOC, which was confirmed with either postoperative MRCP or EUS. Finally, IOC failed in 60/804 patients due to technical difficulties, challenging anatomy or failure of cystic duct cannulation; 29/60 (48%) patients had no further investigations due to normal LFTs whereas the remaining patients underwent MRCP. Of these patients, seven required postoperative ERCP and CBD stone removal.

## Discussion

Routine IOC plays an important role in the identification of CBD stones in the elective, semi-elective and emergency settings. Although the overall detection rate of CBD stones is low (5.8%), confirmed by subsequent ERCP, no patients in our cohort were readmitted with CBD stones. The reported incidence of CBD stones varied between 8-15% [8-11]. Furthermore, preoperative investigations are not predictive of negative IOC with some patients having filling defects on IOC despite nonsuggestive preoperative findings. Moreover, confirmation of stones on ERCP revealed a relatively high false-positive rate although this could be attributed to stones having passed between IOC and ERCP. This data is consistent with other studies showing up to one-third of patients with an abnormal IOC having a normal ERCP [12]. Conversely, up to 57% of patients with a normal IOC can have CBD stones on subsequent ERCP [13]. Our data did not reveal significant predictors of IOC filling defects aside from ALP. Other studies have reported raised bilirubin and advanced age as predictive of filling defects on IOC [13]. Thus, the missing of CBD stones cannot be completely avoided with routine use of IOC, although routine IOC provides the most comprehensive option for detecting them. This further avoids unnecessary ERCP, which is associated with the risk of significant complications [14].

IOC is the gold standard tool to determine the presence of CBD stones as well as to delineate biliary anatomy [5]. IOC has a reported sensitivity and specificity of 99% in the identification of CBD stones [4]. Furthermore, in difficult cholecystectomies where dissection of the hepatocystic triangle is difficult, IOC can prevent or identify CBD injury [15,16]. The routine use of IOC, however, remains controversial when performing laparoscopic cholecystectomies. Routine IOC has the advantage of avoiding the missing of CBD stones not detected on preoperative investigations or those formed in the interval between investigations and eventual operation. Given the delays that often occur in performing timely cholecystectomies, the alternative is to repeat investigations including imaging prior to the operation. The disadvantages of routine IOC include technical difficulties that could increase operative time, costs and risk of post-procedure pancreatitis [7]. Moreover, patients may have false-positive filling defects present, causing unnecessary CBD exploration or ERCP [13]. In addition, selective IOC is advocated in the presence of jaundice, pancreatitis, deranged LFTs, ultrasound evidence of a dilated CBD (>6 mm) or uncertain intraoperative biliary anatomy [6]. Given the high negative predictive value of clinical features, biochemistry and ultrasonography, this is a more rational approach although it is not guaranteed to avoid the missing of CBD stones [3,6]. A systematic review of four trials involving 860 patients found selective IOC to be associated with shorter operative time, fewer complications and comparable risk of CBD injury [17]. However, routine IOC was useful in perioperative CBD stone detection and was associated with a reduced risk of readmission for CBD stones. Thus, despite the added challenges of performing routine IOC, it can be beneficial in preventing recurrent admissions and complications associated with retained stones.

Our study has several limitations. This was a single-centre, single-surgeon retrospective study, and hence it could be prone to the inherent flaws of such studies, including an inability to control outcomes or infer causality. Furthermore, the timings of interventions including ERCP were wide-ranging with some patients having normal ERCPs post-positive IOC, suggesting a stone may have passed. Alternatively, this may be indicative of artefacts in the IOC leading to false-positives. Finally, we have not been able to validate the IOC findings with on-table CBD exploration as this was not routinely performed. As part of the protocol, a postoperative ERCP was preferred. These limitations may be overcome by conducting a prospective multi-centre trial in the furure.

## Conclusions

Based on our findings, the routine use of IOC is safe and effective as it would enable CBD stone detection, which can help plan further interventions. Moreover, due to the delays in performing laparoscopic cholecystectomies following acute admissions, preoperative investigations may not be informative at the time of operation, suggesting a role for routine IOC although this may result in excessive IOCs being performed.
